# Supplementary figures and images for: Essential Oil of Mentha aquatica var. Kenting Water Mint Suppresses Two-Stage Skin Carcinogenesis Accelerated by BRAF Inhibitor Vemurafenib
Source: Molecules. 2019 Jun 25;24(12):2344. doi: 10.3390/molecules24122344 (PMC6630265; doi:10.3390/molecules24122344)

# Supplementary Figure 1

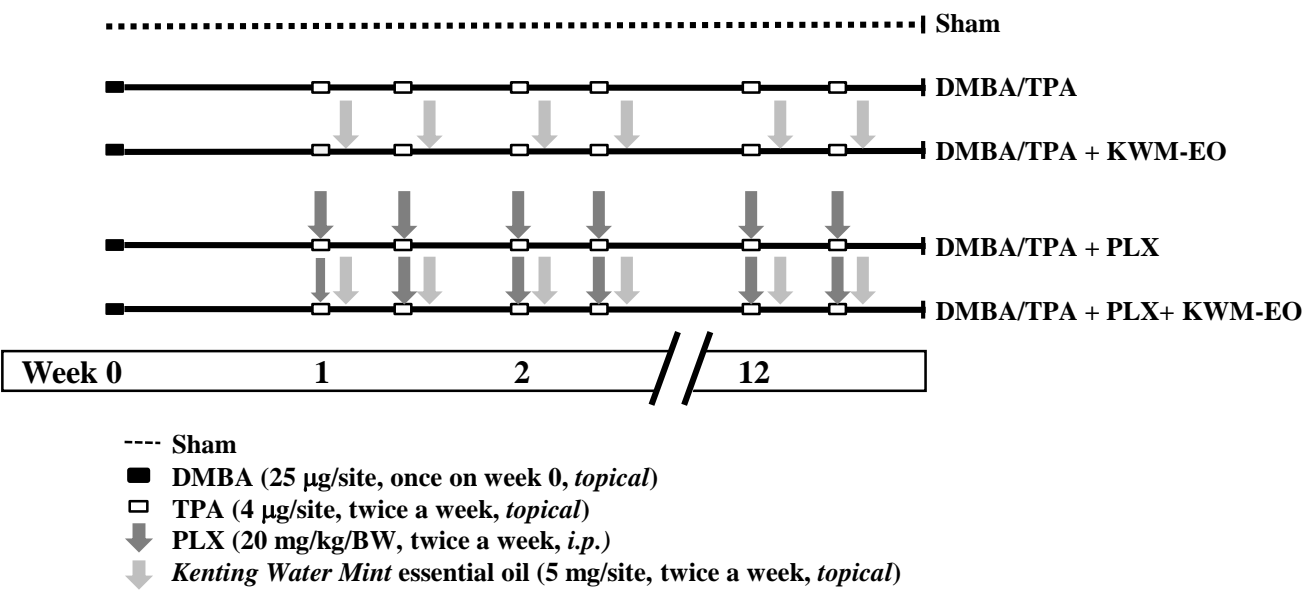

# Supplementary Figure 2

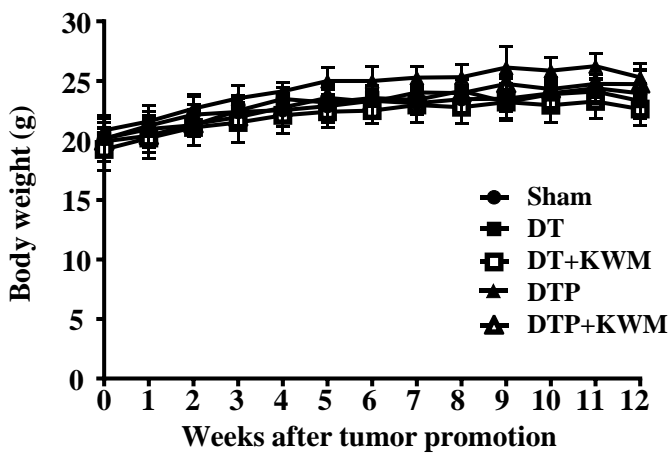

Supplement: Supplementary file 1 [file molecules-24-02344-s001.pdf]
